# Money Walks: Implicit Mobility Behavior and Financial Well-Being

**DOI:** 10.1371/journal.pone.0136628

**Published:** 2015-08-28

**Authors:** Vivek Kumar Singh, Burcin Bozkaya, Alex Pentland

**Affiliations:** 1 Media Lab, Massachusetts Institute of Technology, 20 Amherst St, Cambridge, Massachusetts, United States of America; 2 School of Management, Sabanci University, Tuzla, Istanbul, Turkey; 3 School of Communication and Information, Rutgers University, New Brunswick, New Jersey, United States of America; University of Namur, BELGIUM

## Abstract

Traditional financial decision systems (e.g. credit) had to rely on explicit individual traits like age, gender, job type, and marital status, while being oblivious to spatio-temporal mobility or the habits of the individual involved. Emerging trends in geo-aware and mobile payment systems, and the resulting “big data,” present an opportunity to study human consumption patterns across space and time. Taking inspiration from animal behavior studies that have reported significant interconnections between animal spatio-temporal “foraging” behavior and their life outcomes, we analyzed a corpus of hundreds of thousands of human economic transactions and found that financial outcomes for individuals are intricately linked with their spatio-temporal traits like *exploration*, *engagement*, and *elasticity*. Such features yield models that are 30% to 49% better at predicting future financial difficulties than the comparable demographic models.

## Introduction

An ability to understand and predict financial outcomes for individuals (e.g. their propensity to *overspend*, *miss payments*, or *become delinquent*) is of interest to economists, policy designers, financial institutions, and the individuals themselves. According to the Nilson reports, there were more than 3 billion credit cards in use in 2013, accounting for purchases exceeding US$ 2.2 trillion, and according to the Federal Reserve report, 39% of American households were carrying credit card debt from month to month [1–2]. An individual equipped with better insights can make more effective financial decisions and possibly change some of their habits to reduce these risks. On the other hand, better decision-making capabilities will allow banks to reduce “bad lending,” which was cited as a leading cause of the 1990s American and Asian financial crises [3].

Parallel to the growth trend in mobile devices, internet-of-things, and location-aware payment devices, one of the biggest growth trends in 2014 has been geo-aware mobile payments. According to a recent US Federal Reserve report, 33 percent of mobile phone owners have used mobile banking in the past 12 months and 17% of mobile phone users have made a payment using a mobile phone [4]. According to Forrester, mobile payments are expected to exceed $140 billion in the next 5 years [5]. This growth in mobile payments and relevant computational social science techniques present an opportunity to undertake cross-sectional studies of human shopping behavior across space and time, and understand its associations with financial outcomes [6–9].

While multiple studies have connected an individual’s financial outcomes to her socio-demographic standing or past payment history [10–12], no publicly reported studies have looked at fine-grained data of the individual’s *current* shopping behavior to predict financial outcomes. While multiple reasons could account for this gap, three contributing reasons have been: (1) the industrial secrecy surrounding credit scoring and similar processes; (2) the lack of large scale geo-temporally inscribed data concerning financial transactions; and (3) infrequent or insufficient interactions between the researchers studying biological roots of human mobility behavior and those studying financial outcomes. Hence, in this work we study a large-scale cross-sectional dataset of human spending across space and time, and connect it to the biological phenomena of “foraging,” a basic pattern of animal movement to gather foods and resources.

A rich body of literature on animal ecology has shown that different animals exhibit species-specific patterns of foraging [13–20]. When presented with a spatial distribution of resource “patches” with different utility, most animals increase the proportion of their foraging time spent on the high-utility patches. However the degree of “focus” varies between animals and there is always a trade-off between the conflicting demands of sampling a variable environment and the exploitation of the most useful resources [13–14]. The response of different animals to this trade-off varies and while some animals demonstrate higher degrees of **“exploration”** across patches, others tend to show higher degrees of **“exploitation”** of the known patches [13–17]. Further, while certain animals tend to show very similar patterns of behavior over time even when there are changes in the environment, others demonstrate behavioral **“plasticity”** and change their behavior promptly [19–20].

A combination of these behavioral traits—exploration, exploitation, and plasticity—has been demonstrated to have significant predictive power on an animal’s social stature, survivability, metabolism rates, reproductive success, and other life outcomes [15–20]. Our analysis suggests that similar traits show significant predictive power on financial outcomes for human beings. Specifically, measuring these three behavioral traits yields models that are **30% to 49% better than comparable demographic models for predicting financial outcomes.**

This makes sense, as behind our veneer of language, culture, and social norms, human beings are still biological animals. Focusing on intrinsic behavioral traits shared with other animals also allows us to focus on aspects that may have evolved before speech or higher level social constructs. Thus the results obtained may have a higher likelihood of being consistent across cultures than those based on higher level constructs such as culture-specific norms and customs.

Current financial analysis systems focus mainly on explicit demographic traits provided by the customer, leaving room for advertent (fraud) and inadvertent (ambiguity, sparsity) data issues [21–22]. For example, a malicious individual may fake their job-type, or a micro-loaning establishment in a developing country may not be able to obtain accurate dates of births for its new clients. However, the spatio-temporal behavioral markers obtained directly from the shopping data provide more transparent clues to individual financial habits. Such mobility markers may also be harder to manipulate than a social or economic profile or recent payment history [23]. In the future, this may allow individuals to demonstrate their credit-worthiness based on mobility traces rather than financial statistics or property-based collaterals.

## Materials and Methods

### Data

The study was approved by the Sabanci University Research Ethics Council. Details: Customer name and unique identifier (such as the equivalent of a Social Security Number) were removed from the sample before analysis. The analysis reported in this paper was undertaken on this anonymized and de-identified dataset. Such individual level data concerning demography, behavioral features, and financial outcomes (true, false) along with the appropriate analysis code has now been made publically available for replication at http://bit.ly/1vNAQYL.

The considered database consists of tens of thousands of individual accounts (approximately 10% of all customer accounts) sampled from a large financial institution’s data warehouse in an OECD country. For each customer account sampled, all credit card transactions for purchases made within a 3-month window in 2013 were collected. This amounts to about 15.7 million transactions for a total of 405 thousand customer accounts. The sampling period was selected to avoid a special shopping occasion (e.g. festive season or major holidays) that could possibly bias the data collected.

The transaction data we sampled include the following attributes regarding the purchases made as well as certain demographic features of the customer(s) who made the purchases:

- Timestamp for transaction (date, hour and minute)
- Transaction amount
- Merchant address (where the transaction took place)
- Merchant XY coordinates
- Merchant spending category
- Customer home and work address
- Customer home and work XY coordinates
- Customer age
- Customer gender
- Customer marital status
- Customer education level
- Customer’s income, as estimated by the bank

The customer-related data were anonymized by excluding customer name or unique identifier (such as the equivalent of a Social Security Number) from the sample, and also by associating a pseudo-unique number with each customer for back-tracking by the bank officials. The attributes listed above are further processed into other calculated measures or indices, as described below that are used in the analyses reported in this paper.

### Features

To analyze the relationship between individuals’ spending behavior and their corresponding financial well-being, we have devised several measures or indices. The spatio-temporal behavior of each customer was measured based on three features, which were each computed with four variations based on the definition of the “bins”:

- Diversity: this refers to the fact that a customer’s shopping experience may vary significantly over time and space. This means that a customer spreads his transactions equitably over various “bins”.
  The “bins” have been defined as segments over space and time (further explained below). Given the bin definition, we calculate for each bin *p*_*ij*_, the fraction of transactions that fall within bin *j* for user *i*. The spatial diversity of user *i* is then calculated as the normalized entropy of all transactions counted in all *N* bins with *M* of such bins being non-empty, and is given by:
  $$D_{i}=\frac{-\sum_{j=1}^{N}p_{ij}\text{log}p_{ij}}{\text{log}M}$$ (1)
  The resulting values are between 0 and 1, with the larger numbers meaning higher spatial diversity. For example, a user with very high spatial diversity spread his/her transactions almost equitably across different locations. The normalization by log *M* keeps the focus on quantifying the relative *spread* across bins, and gives equitable chance for customers with different number of transactions (and occupied bins) to score high on this measure.
- Loyalty: loyalty characterizes the percentage of transactions that happen over a customer’s “k” most frequently used “bins”. Given the same definition of bins, we consider the top 3 most-frequented bins for each user. Let *f*_*i*_ be the combined fraction of all transactions of user *i* that occur in the top 3 most-frequented bins. The loyalty of user *i* is then calculated as follows:
  $$L_{i}=\frac{f_{i}}{\sum_{j=1}^{N}p_{ij}}$$ (2)
  The resulting values are also between 0 and 1, with the larger numbers meaning higher spatial loyalty. For example, a user with very high spatial loyalty concentrated most of her transactions within top-three of her visited locations.
- Regularity: Regularity measures the level of similarity in user’s behavior over shorter (1 month) and longer (3 month) term periods. Specifically, we use ***SD***_***i***_ and ***SL***_***i***_ vectors and calculate the normalized Euclidean distances between them. The resulting temporal regularity index of user *i* is calculated as:
  $$R_{i}=1-\frac{\sqrt{{\left(D_{i}^{1}-D_{i}^{T}\right)}^{2}+{\left(L_{i}^{1}-L_{i}^{T}\right)}^{2}}}{\sqrt{2}}$$ (3)
  where $D_{i}^{1}$ and $D_{i}^{T}$ refer to the diversity of the user in the first month and in the entire period, respectively. Similarly, $L_{i}^{1}$ and $L_{i}^{T}$ correspond to the spatial loyalty of the user in the same time frames. The resulting distance values ***R***_***i***_ are between 0 and 1, with 1 indicating perfect regularity (i.e. the two vectors are identical, and hence the first month’s and overall spending patterns are) and 0 indicating perfect irregularity.

#### Definition of “bins”

The bins were defined based on two interpretations for space and two for time.

- Spatial-Grid: a square grid structure where each grid cell is 0.1 by 0.1 decimal degree units.
- Spatial-Radial: a radial structure where bins are concentric circles that have 1, 3, 5, 15, 50, 150, and 500 kilometer radii, respectively, around home and work locations of the customer.
- Temporal-Hourly: hour of the day. For example, (8 am-9 am) was one bin and (9 am -10 am) another.
- Temporal-Weekly: day of the week. For example, Monday was one bin and Tuesday another.

#### Characterizing financial well-being outcomes

On the dependent variables side, we consider the following variables/indicators:

- Overspending: We keep track of each customer’s level of overspending by comparing total credit card transactions for the analysis period with the customer’s total income as estimated by the bank. Let *cc*_*i*_ be the amount spent by customer *i*, and let *I*_*i*_ be her estimated total income. The overspending variable for customer *i* is calculated as: $O_{i}\;=\;\frac{{cc}_{i}}{I_{i}}$ For an overspending customer who spends more than the earned income, this ratio should be over 1, and the higher the ratio is, the more “overspending” and hence financially risky the customer is.
- Trouble: This variable is a 0–1 variable where 1 simply indicates that the customer has either defaulted during the analysis period or experienced financial trouble as detected by the bank. The bank keeps track of the customer’s status in three levels of financial trouble. The lowest level indicates that the customer has not paid or was significantly late in his/her credit card statement. The second and next serious level of detail indicates that the bank has taken an administrative action with the customer who has not paid or paid late a number of times in recent history. The third and most serious level of financial trouble is that the customer can no longer pay back his/her balance and a legal action has been started. The Defaulting variable is based on all of these three levels of financial trouble; i.e. the variable takes the value of 1 if any of the three scenarios are observed for a customer. We further consider an additional period of 3 months immediately following the analysis period, mainly because the signs of financial trouble may be observed with a lag after the individual transactions take place.
  $$D_{it}=\{\begin{array}{c} 1,\;\text{ if the customer experienced trouble in the analysis period}\\ 0,\;\text{otherwise} \end{array}$$ (4)
- Late Payment: This variable indicates whether the customer paid his/her credit card balance late. Since the analysis period includes more than one statement and possibly multiple payments, we considered the combined total number of late days for all statements and payments. One has to be cautious though when using this variable, as a late payment may be due to reasons that are unrelated to financial trouble (such as traveling out of town and missing the deadline, or simply forgetting to pay). In our analysis, we accounted for this possibility by using a grace period of 3 days and re-running our models, which nevertheless did not significantly change the results we have reported in the previous section.

A summary of the features defined is presented in Table 1.

**Table 1:** A summary of various behavioral and financial outcome features considered in this work and their descriptive statistics.

**Behavioral features defined**		
**Hourly_diversity**	**Hourly_loyalty**	**Hourly_regularity**
Min.: 0.3922	Min.: 0.2400	Min.: 0.1628
1st Qu.:0.9035	1st Qu.:0.4000	1st Qu.:0.8061
Median: 0.9280	Median: 0.4706	Median: 0.8943
Mean: 0.9203	Mean: 0.4878	Mean: 0.8520
3rd Qu.:0.9477	3rd Qu.:0.5556	3rd Qu.:0.9409
Max.: 1.0000	Max.: 1.0000	Max.: 0.9992
**Weekly_diversity**	**Weekly_ loyalty**	**Weekly_ regularity**
Min.: 0.4879	Min.: 0.4473	Min.: 0.2247
1st Qu.:0.9083	1st Qu.:0.5818	1st Qu.:0.8220
Median: 0.9405	Median: 0.6389	Median: 0.8957
Mean: 0.9296	Mean: 0.6553	Mean: 0.8645
3rd Qu.:0.9646	3rd Qu.:0.7143	3rd Qu.:0.9452
Max.: 1.0000	Max.: 1.0000	Max.: 1.0000
**Spatial.radial_ diversity**	**Spatial.radial_loyalty**	**Spatial.radial_ regularity**
Min.: 0.0000	Min.: 0.4286	Min.: 0.2405
1st Qu.:0.7188	1st Qu.:0.8182	1st Qu.:0.8209
Median: 0.8292	Median: 0.9091	Median: 0.8946
Mean: 0.7652	Mean: 0.8916	Mean: 0.8313
3rd Qu.:0.9069	3rd Qu.:1.0000	3rd Qu.:0.9440
Max.: 1.0000	Max.: 1.0000	Max.: 1.0000
NA's: 824	NA's: 824	NA's: 1137
**Spatial.grid_diversity**	**Spatial.grid_loyalty**	**Spatial.grid_ regularity**
Min.: 0.0000	Min.: 0.2857	Min.: 0.1532
1st Qu.:0.6485	1st Qu.:0.7907	1st Qu.:0.6434
Median: 0.7897	Median: 0.9048	Median: 0.7504
Mean: 0.7283	Mean: 0.8728	Mean: 0.7255
3rd Qu.:0.8860	3rd Qu.:1.0000	3rd Qu.:0.8448
Max.: 1.0000	Max.: 1.0000	Max.: 1.0000
NA's: 33	NA's: 33	NA's: 409
**Demographic features**		
**Age**	**Gender**	**Marital Status**
Min.: 18.00	Female:2407	Divorced: 458
1st Qu.:30.00	Male: 6604	Married: 5970
Median: 36.00		Single: 2581
Mean: 37.65		Unknown: 2
3rd Qu.:44.00		
Max.: 91.00		
**Education**	**Type of Work**	
College: 3324	Private Sector Employee: 6160	
High School: 3470	Public Servant: 1657	
Master: 304	Retiree & Non-Employed: 382	
Middle School: 802	Self-Employed—Service: 289	
PHD: 29	Self-Employed—Retail: 130	
Primary School:1079	Self-Employed—Manufacture: 128	
Unknown: 3	(Other): 265	
**Financial outcome variables**		
**Late Payment**	**Overspending Classification**	**Financial Trouble**
Min.: 0.0000	Min.: 0.0000	Min.: 0.0000
1st Qu.:0.0000	1st Qu.:0.0000	1st Qu.:0.0000
Median: 0.0000	Median: 0.0000	Median: 0.0000
Mean: 0.3191	Mean: 0.4999	Mean: 0.1291
3rd Qu.:1.0000	3rd Qu.:1.0000	3rd Qu.:0.0000
Max.: 1.0000	Max.: 1.0000	Max.: 1.0000

#### Data characteristics/cleaning/validation

**Fig 1:**
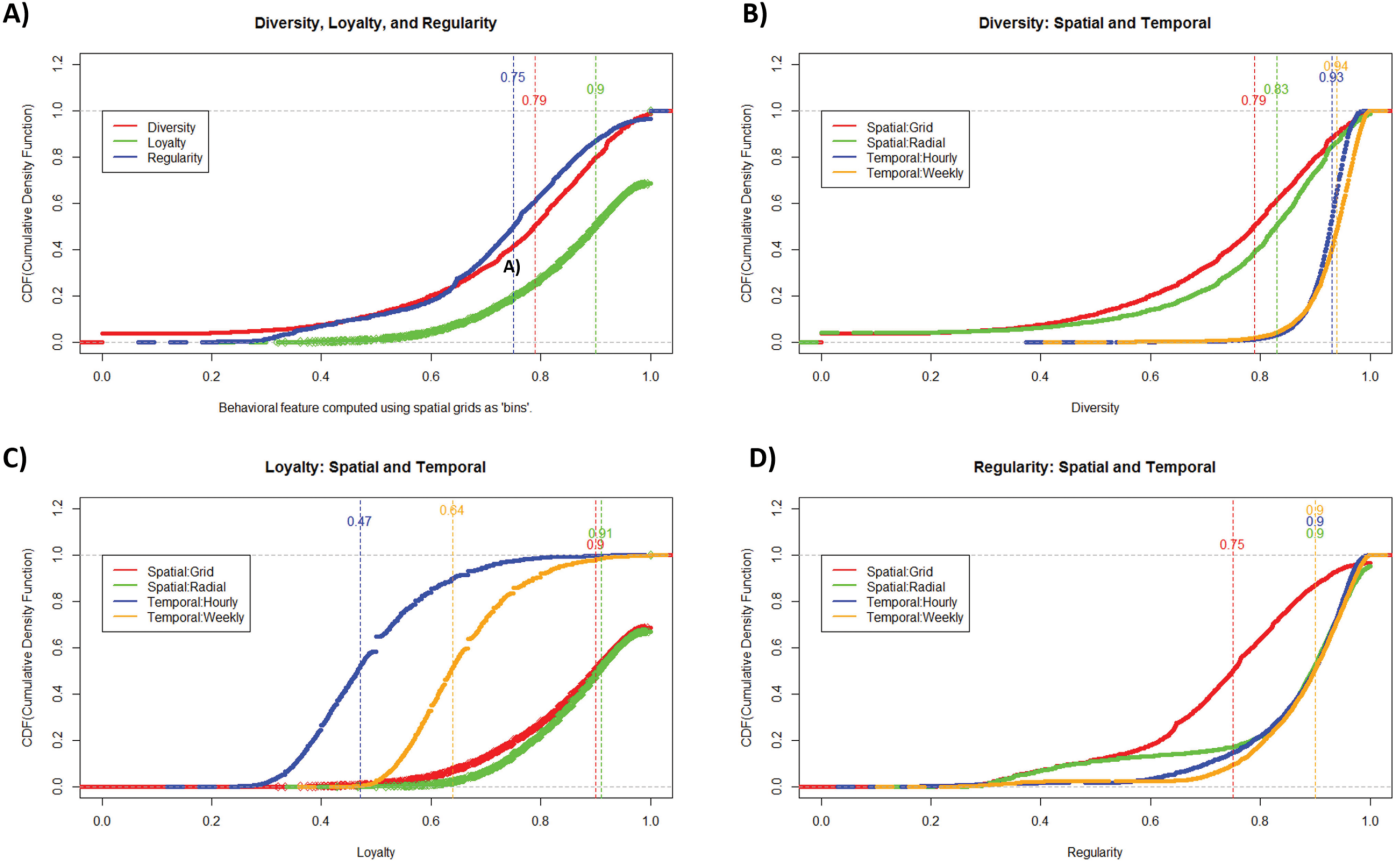
Cumulative density functions (cdf) of for diversity, loyalty, and regularity exhibited by the customers. A) All three curves have high median scores, thus indicating a strong affinity for all three traits in human shopping behavior; B) cdf for *diversity*, computed over space and time, shows that the customers were a lot more diverse in terms of their times of shopping than the locations visited; C) cdf for *loyalty*, computed over space and time, shows that the customers’ three most preferred locations account for a very large proportion (~90%) of all their shopping; and D) cdf for *regularity*, computed over space and time shows that most users exhibit very similar behavioral patterns over time. However, about 10% to 25% of the users exhibit low regularity, which could be useful for identifying anomalous behavior as well as rarer financial outcomes like delinquency.

**Fig 2:**
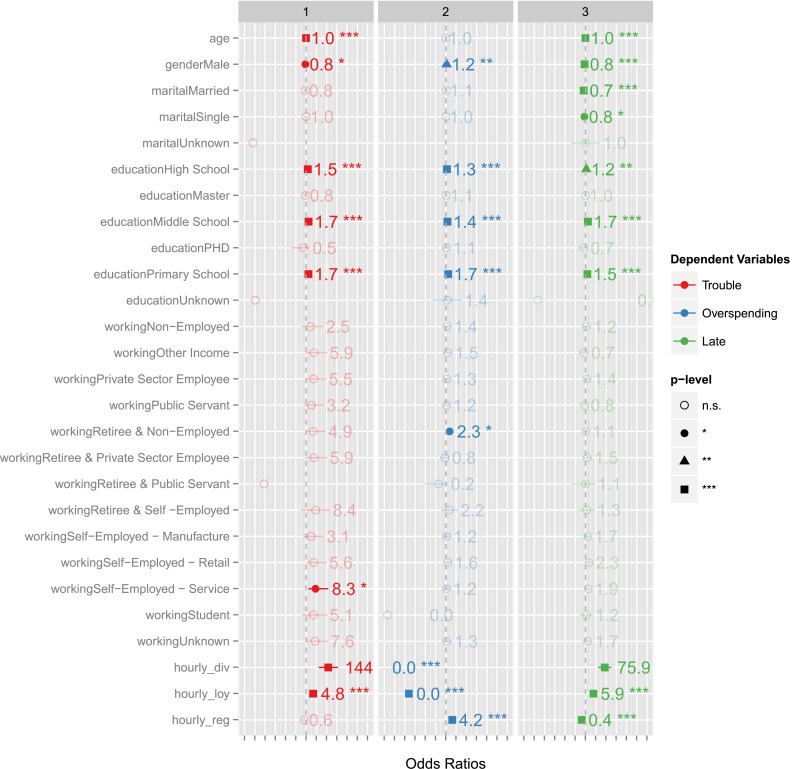
(A) Significant coefficients observed based on logistic regression between the financial outcomes and the demographic and behavioral features. Individuals with lower levels of education (High School, Middle School, or Primary School) are more likely to be late for their payments and get into financial trouble. Users with higher age were marginally less likely to overspend, miss payments, or get into financial trouble. Last, male customers and married customers were less likely to miss their payments. The behavioral mobility features *(diversity*, *loyalty*, and *regularity* computed based on hours as bins) were found to be significantly associated with the considered financial outcomes. Individuals who are “regular” were more likely to pay their bills on time. Users with a high degree of diversity or loyalty were less likely to overspend, yet more likely to miss payments or get into financial trouble. In relative terms, the behavioral features were found to be more significantly associated (in terms of p-values) and contain higher predictive power (in terms of odds ratios being further away from 1.0 in either direction) as compared to the demographic features.

**Fig 3:**
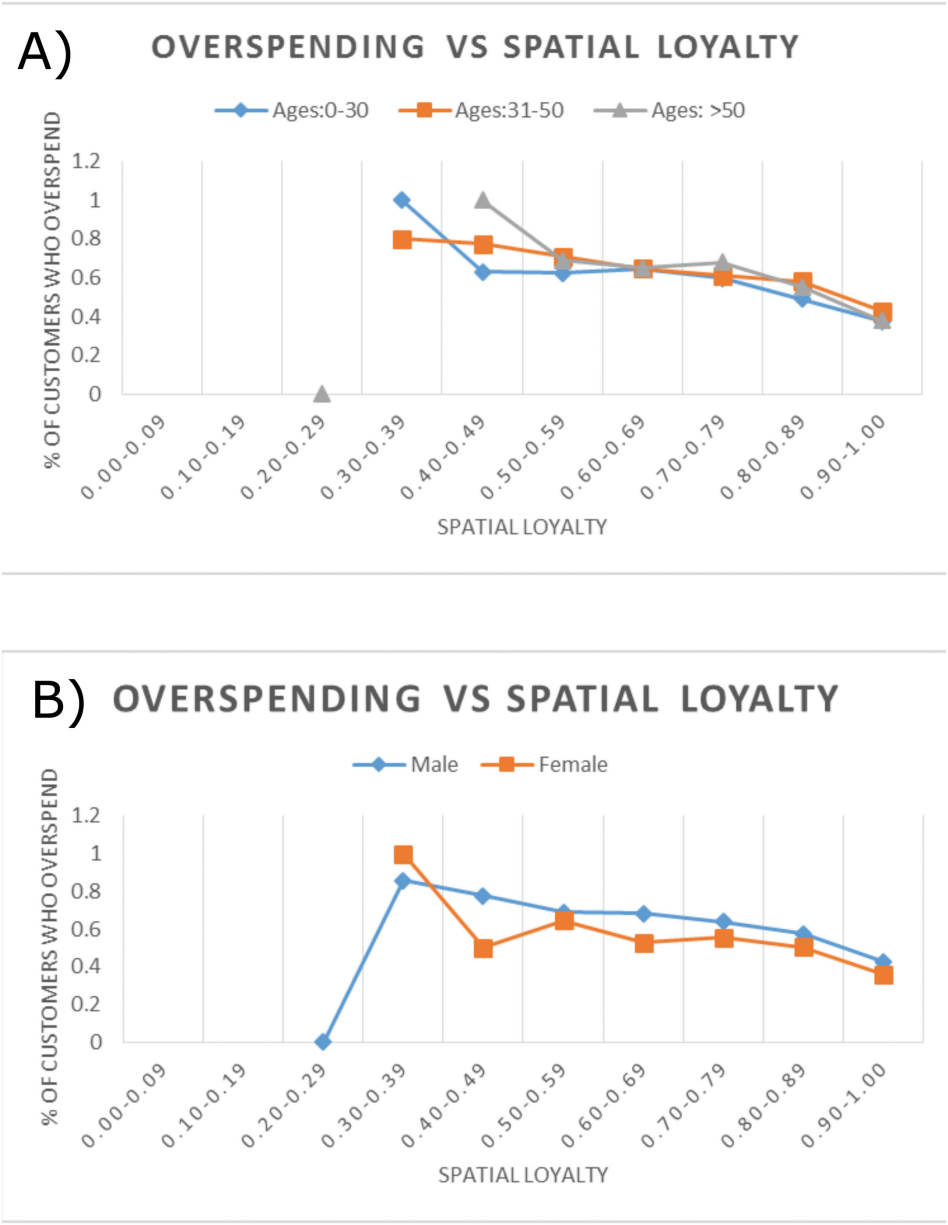
The relationship between loyalty and overspending, as demonstrated across individuals belonging to A) different age groups and B) gender. Customers with lower spatial loyalty are more likely to overspend across different demographic “bands” of age and gender, thus indicating a general pattern connecting overspending and spatial loyalty. There is one outlier point in each of the two yfigures corresponding to a bin (spatial loyalty 0.20–0.29), in which there was only one customer.

**Fig 4:**
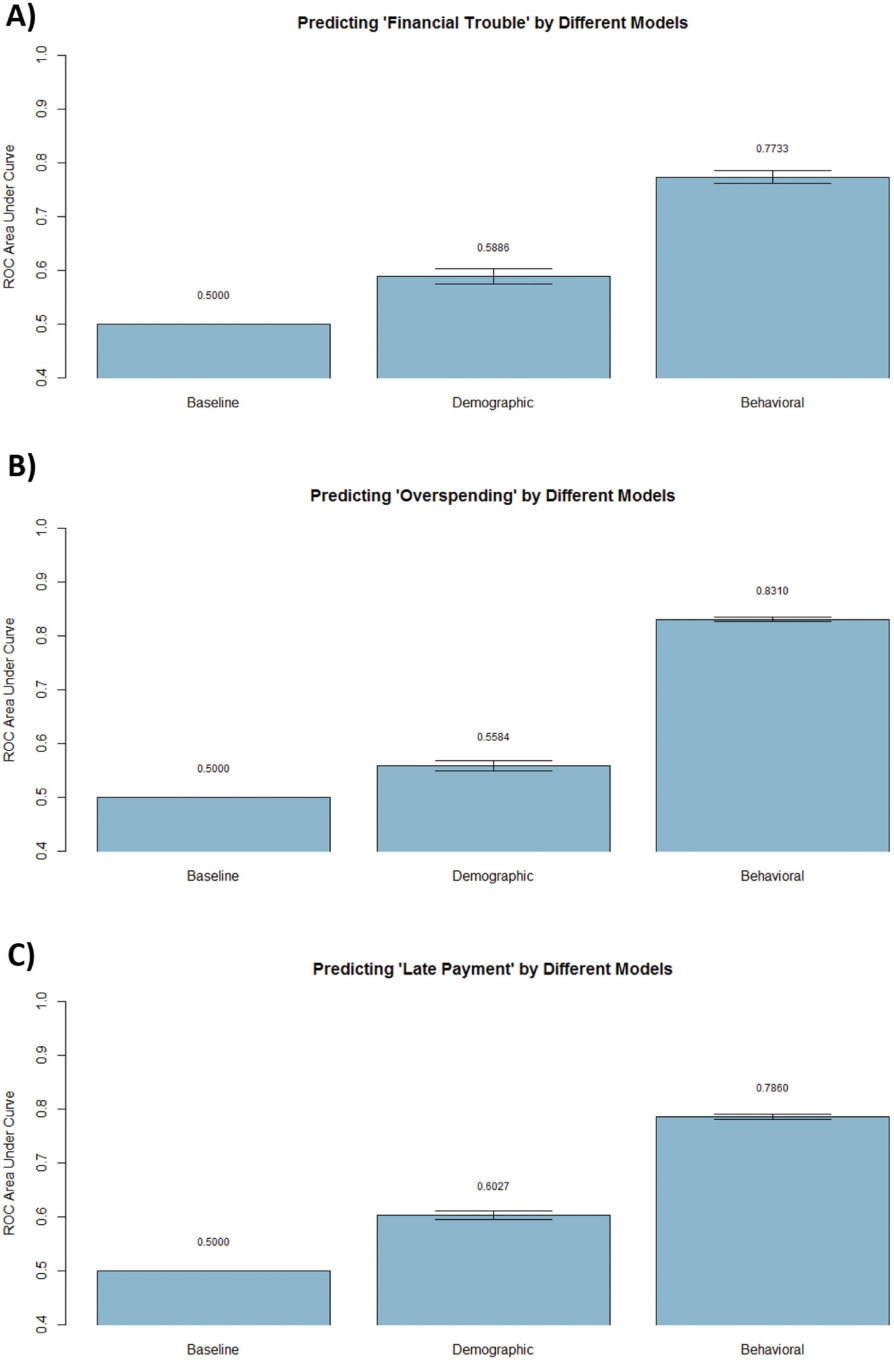
Prediction performance for different financial outcomes using a baseline, demography-based, and behavior-based model. The behavioral models perform 31%, 49%, and 30% better than the corresponding demography models for predicting “financial trouble", "overspending", and "late payment", respectively.

We selected a random sample of 10,000 individuals from the bank database for this study (number of transactions = 386,610). We excluded online transactions from this dataset. We considered individuals with less than 10 transactions in the three months as “inactive”, and excluded them from the analysis yielding a subset of 9,011 individuals. Also, we excluded the home/work and shopping location addresses that were not geo-coded, which resulted in complete set of features being available for 7,855 users. The cumulative density function, odds ratio, and analysis across demographics bands (Figs 1–3 in the Results and Discussion section) are based on 7,855 users. The machine learning approach adopted is considered more robust to missing data and hence the classification results (Fig 4) are based on 9,011 users.

#### Classification Method

The algorithm used for classification is Bagging (Bootstrap Aggregation) [24] that was run with subset evaluation based feature selection [25]. We used 70% of the data as training data set and the remaining 30% were used as the testing data set. The classification results reported are based on 30 rounds of classification run with resampling (with replacement) and a different randomized seed for each round. All implementations of these algorithms were used as implemented in Weka software (version 3.6.12) available from http://www.cs.waikato.ac.nz/ml/weka/.

The datasets considered in this work were not well-balanced. For example, 13 percent of customers were in some kind of financial trouble, while 87 percent were not. To mitigate the effects of such imbalance, we applied the `SMOTE' approach to create a balanced data set for training, before testing in the realistic imbalanced settings. SMOTE (Synthetic Minority Oversampling TEchnique) works by under-sampling the majority class and over-sampling the minority class. However, it mitigates the problem of over-fitting caused by simple replication of data points by generating newer (synthetic) examples by operating in ‘feature space’ rather than ‘data space’. Please see [26] for a detailed discussion.

## Results and Discussion

The current study is undertaken using a de-identified credit card transaction database from a major financial institution. The analysis of spatio-temporal trails for 10,000 randomly selected customers (number of transactions = 386,610) revealed that multiple spatio-temporal features were significantly associated with financial outcomes.

We consider three main output variables to measure individual financial well-being, namely **overspending**, **late payment,** and being in “**financial trouble**” as documented by an administrative action of the bank. To construct the behavioral profile for each individual, we characterize their spatio-temporal spending trail using metrics for **diversity, loyalty,** and **regularity** as proxies for the behavioral traits of **exploration**, **exploitation**, and **plasticity** respectively.

Each of the traits of diversity, loyalty, and regularity was defined similarly in both space and time. For example, spatial diversity captures the spread of transactions over different locations; loyalty characterizes the percentage of transactions that happen over a customer’s “k” most frequently visited locations, and regularity is the similarity of behavior over short and long periods of time. Similar definitions were applied to define these properties over temporal bins where bins were defined as: (1) *hours of the day* and (2) *days of the week*. (See Materials and Methods section.)

Fig 1A illustrates the cumulative density function (cdf) of spatial diversity, loyalty, and regularity for the customers analyzed. We notice that all three curves have high median scores, thus indicating a strong affinity for all three traits in human shopping behavior. Customers who tend to shop at diverse locations but at the same time have certain “preferred locations” that account for a large portion of their purchases. Further, most people exhibit very similar shopping behavior over time.

Fig 1B, 1C and 1D show a comparison of the three behavioral metrics across both space and time. Fig 1B shows that the customers were a lot more diverse in terms of their times of shopping than the locations visited. Similarly, Fig 1C shows that the customers’ three most preferred locations account for a very large proportion (~90%) of all their shopping. By contrast, users made less than 65% of their purchases in their most preferred timeslots. Last, in terms of regularity, Fig 1D shows that most users exhibit very similar behavioral patterns over time. However, about 10% to 25% of the users exhibit low regularity, which could in turn be useful for identifying anomalous behavior as well as rarer financial outcomes like delinquency.

A regression analysis of the financial outcome variables reveals that multiple demographic and behavioral variables are significantly associated with them. (The significance was based on the p-value of the coefficients in the generalized linear model for logistic regression. Given multiple comparisons, we focus on coefficients that are significant after Bonferroni corrections.) As shown in Fig 2, individuals with lower levels of education (High School, Middle School, or Primary School) were found to be more likely to be late for their payments and get into financial trouble. Users with higher age were marginally less likely to overspend, miss payments, or get into financial trouble. Last, male customers and married customers were less likely to miss their payments.

The figure also shows that multiple mobility behavior features were statistically correlated with outcome variables, *even after controlling for the effect of abovementioned demographic variables of age*, *gender*, *marital status*, *education*, *and work type*. The coefficients for diversity, loyalty, and regularity were found to be significantly associated with the considered financial outcomes. For example, the individuals who are “regular” in their behavior also tend to pay their bills on time. In contrast, the users with a high degree of diversity or loyalty were less likely to overspend, yet more likely to miss payments or get into financial trouble.

In relative terms, the behavioral features were found to be more significantly associated (in terms of p-values) and contain higher predictive power (in terms of odds ratios being further away from 1.0 in either direction) as compared to the demographic features. While Fig 2 defines the behavioral features using “hours” for binning, very similar trends were obtained when the behavioral features were defined over days of the week (temporal), spatial radials, and spatial bins. Similar figures obtained for other binning methods are shown as supplementary material S1, S2, S3 Figs and a summary of the associations observed between behavioral features and financial outcomes is presented in Table 2.

**Table 2:** A summary of the significant associations found between behavioral traits defined over space and time and financial outcomes. Behavioral features were found to be significantly associated with financial outcomes even with varying approaches to define spatio-temporal bins, thus indicating a general pattern connecting financial outcomes and mobility behavior.

	Trouble		Overspending		Late Payment	
**Hourly_diversity**	144.7244	***	4E-07	***	75.9443	***
**Hourly_loyalty**	4.836349	***	0.00025	***	5.90618	***
**Hourly_regularity**	0.644408		4.19918	***	0.42831	***
**Weekly_diversity**	14.87973	*	0.01631	***	3.8659	.
**Weekly_loyalty**	8.457047	***	0.00167	***	7.15294	***
**Weekly_regularity**	0.428613	**	14.0527	***	0.34253	***
**Spatial_radial_diversity**	1.1042		0.69378	**	1.15596	
**Spatial_radial_loyalty**	5.418137	***	0.01341	***	3.07622	***
**Spatial_radial_regularity**	0.557091	**	3.18449	***	0.51707	***
**Spatial_grid_diversity**	1.503061	*	0.36159	***	1.35042	*
**Spatial_grid_loyalty**	4.044549	***	0.01668	***	1.63829	*
**Spatial_grid_regularity**	0.579141	*	1.44744	*	0.59842	**

Significance codes are as follows: 0 ‘***’ 0.001 ‘**’ 0.01 ‘*’ 0.05 ‘.’ 0.1 ‘ ‘ 1

To further interpret these phenomena, we studied if the 12 behavioral features were independent or if they could be reduced into some higher order traits. As shown in S4 Fig, a hierarchical clustering of the features yielded four higher dimensional traits that matched quite closely with the posited axes of diversity, loyalty, and regularity. The exception was the week-day based diversity score which fell into its own separate cluster. A Principal Component Analysis on the 12 features also pointed to a smaller dimension (4), which captured most of the variance in the signal. Last, a correlation analysis between the three outcome variables (overspending, trouble, and late payment) did not yield any statistically significant associations.

To further identify the effect of behavioral traits over and above the demography variables, we studied the effect of different behavioral features across diverse “bands” of individuals. For example, Fig 3 illustrates the interaction between overspending, loyalty (spatial-grid), and two demographic variables (age and gender). For such analysis customers were binned into ten equal sized categories based on their spatial loyalty score. For each bin, the percentage of customers with higher than median overspending ratio is shown in the figure. Consistent with Fig 2 we observe that customers with lower spatial loyalty are more likely to overspend. Interestingly this trend stays consistent across different “bands” of individuals: those in different age groups (Fig 3A) and those of different gender (Fig 3B). These figures motivate the value of behavioral features in conjunction with, rather than in lieu of, demographic variables for predicting different financial outcomes.

The evidence so far indicating that each of the spatio-temporal behavioral descriptors has significant association with different financial outcomes motivates their combination to predict the financial outcomes. Each of the three outcome variables—overspending, trouble, and late payment—was predicted based on three different models using a **Bag**ging (**B**ootstrap **Ag**gregation) approach. Given the relative imbalance in datasets (e.g. 13% of customers were in financial trouble and 87% were not), the AUC metric (area under the receiver-operating-characteristic curve) was chosen for performance evaluation rather than the conventional accuracy measure. The traditional accuracy measure is not a good metric when the classes are imbalanced and/or the cost of misclassification varies dramatically between the two classes (34). For example, in the considered scenario a baseline classifier (ZeroR) that classifies all users as “not having any financial trouble” will achieve 87% accuracy on the prediction but would not serve as a useful detector of financial trouble in practice. An AUC metric measures the area lying under a receiver operating characteristic (ROC), or ROC curve, which is a graphical plot that illustrates the performance of a binary classifier system as its discrimination threshold is varied [27]. The curve is created by plotting the true positive rate (TPR) against the false positive rate (FPR) at various threshold settings. Such an AUC metric that focuses on a trade-off between TPR and FPR would give such a baseline Zero-R method a score of 0.5, which can be used to quantify the relative gains obtained by other methods.

A comparison of the performance of different models (baseline, demography-based, and behavior-based) is shown in Fig 4. For all three financial outcome variables, the behavioral model significantly outperformed the demography-based model, which in turn outperformed the baseline model. For example as shown in Fig 4A, the behavioral model for predicting financial trouble got an AUC score of 77.33% (0.95 confidence interval: 78.53%, 76.12%) as compared to demography model which obtained 58.86% (0.95 confidence interval: 59.33%, 56.65%) and a baseline model which scored 50.00%. Thus on a relative scale, the behavioral model performed 54.66% better than the baseline and 31.48% better than the demography-based model for predicting “financial trouble”. As shown in Fig 4B and 4C, the behavior-based model consistently outperformed the other two models and yielded AUC scores of 83.10% (0.95 confidence interval: 83.54%, 82.65%) for predicting overspending, and 78.60% (0.95 confidence interval: 79.08%, 78.13%) for predicting late payment. Thus the behavioral models perform 31%, 49%, and 30% better than the corresponding demography models for predicting “financial trouble", "overspending", and "late payment", respectively.

## Discussion

The obtained results highlight the importance of spatio-temporal mobility features in predicting the financial outcomes of individuals. This approach is fundamentally different from those often employed in current state-of-the-art methods. Most financial institutions use *capacity*, *capitals* or *collaterals* (e.g. property owned, reserve cash, debt-to-income ratio) which are static, one-time data to estimate the credit-worthiness of a customer, or use segmentation approaches which put many individuals into one unified bucket (e.g. based on age, gender, or educational qualifications) [10–12, 28–29]. The emergence of individual transaction profiles for each customer now allows for creation of rich personalized models of each user’s behavior that can be used to predict their behavior. Also we note that the kind of analysis described here can be done incrementally during the month before the payment deadlines, thus allowing preemptive remedies *before* a user starts missing her payments and becomes delinquent.

The improvements over the comparable demographic models by the behavioral models for different financial outcomes have ranged from 30% to 49%. We consider these to be highly significant given that they can impact a trillion dollar market segment of credit card limit decisions and credit repayment. The analysis has shown that behavioral factors have a higher predictive power than the well-established demographic features. Our results also corroborate recent findings on human mobility patterns being highly unique [30–32] and largely consistent over time [8], often occurring within the home range [20], and being intricately connected with socio-demographic variables [8, 33]. They also underscore the utility of employing a foraging lens to studying human financial behavior [34].

The use of space and time data to compute behavioral descriptors allows for the use of intuitive and well-understood behavioral markers. With the geo-coding of more and more financial data (ATMs, POS machines, customer and business addresses) and better understanding of user’s mobility (GPS coordinates or cell tower-based locations) as they go about their daily business, we expect the spatio-temporal data available for creating such profiles to only get richer and more robust.

These behavior-to-outcome connections also have implications for the privacy of users [32]. The obtained results show the deep interconnections between human mobility data and financial outcomes. Our hope is that the results presented here will raise user awareness on the implications of sharing mobility data. For example, according to a recent industry report, 42% of mobile apps request access to GPS location data [35] and many of these apps (e.g. a flashlight [36]) may not even need this information for their intended purpose. While there are reasonably explicit guidelines regarding the use of demographic variables for banking and loan purposes (e.g. The Fair Housing Act of 1968 [37]), and online data (White House’s proposed 2012 Privacy Bill of Rights [38]), there is no such awareness or a set of guidelines about the use of mobility data. The findings of a public study like this one are critical to motivating a discussion on the right policy parameters surrounding mobility and behavioral data.

Finally, with the appropriate checks and balances in place, the observations presented here could be used in the future to provide feedback and nudges to the individuals themselves. For example, a sudden increase in concentration of location along with increased diversity in time, e.g., becoming irregular at maintaining the basic rhythms of life (food, work, etc.), could be used to alert the user that they are at higher risk of missing their payments or getting into financial trouble. Of course the final decision about behavior change must always remain with the user: they may choose to ignore the message or use it as a reminder to moderate their behavior.

## Supporting Information

S1 Fig Significant coefficients observed based on logistic regression between the financial outcomes and the demographic and behavioral features. Behavioral features included in this model are diversity, loyalty, and regularity defined using spatial grids as the bins. Significance codes are as follows: 0 ‘***’ 0.001 ‘**’ 0.01 ‘*’ 0.05 ‘.’ 0.1 ‘ ‘ 1.
(EPS)

S2 Fig Significant coefficients observed based on logistic regression between the financial outcomes and the demographic and behavioral features. Behavioral features included in this model are diversity, loyalty, and regularity defined using spatial radials as the bins. Significance codes are as follows: 0 ‘***’ 0.001 ‘**’ 0.01 ‘*’ 0.05 ‘.’ 0.1 ‘ ‘ 1.
(EPS)

S3 Fig Significant coefficients observed based on logistic regression between the financial outcomes and the demographic and behavioral features. Behavioral features included in this model are diversity, loyalty, and regularity defined using days of the week as the bins. Significance codes are as follows: 0 ‘***’ 0.001 ‘**’ 0.01 ‘*’ 0.05 ‘.’ 0.1 ‘ ‘ 1.
(EPS)

S4 Fig Correlation among the behavioral features and their clustering. The Pearson’s correlation scores for pairs of features ranging between +1 and -1 are shown using the color and the size of the circle. Clusters based on hierarchical clustering method are shown as rectangles. Other than the exception of weekly diversity, the features of regularity, diversity, and loyalty form clearly identifiable clusters. This suggests that there is high correlation between the features as measured through different variants of space and time, and the different features capture independent facets of the human spending behavior.
(EPS)
